# Noise and Dynamical Synapses as Optimization Tools for Spiking Neural Networks

**DOI:** 10.3390/e27030219

**Published:** 2025-02-21

**Authors:** Yana Garipova, Shogo Yonekura, Yasuo Kuniyoshi

**Affiliations:** Laboratory for Intelligent Systems and Informatics, University of Tokyo, Tokyo 113-0033, Japan; yonekura@isi.imi.i.u-tokyo.ac.jp (S.Y.); kuniyosh@isi.imi.i.u-tokyo.ac.jp (Y.K.)

**Keywords:** spiking neural networks, adaptability, noise, stochastic resonance, dynamical synapse, leaky integrate-and-fire neuron

## Abstract

Standard ANNs lack flexibility when handling corrupted input due to their fixed structure. In this paper, a spiking neural network utilizes biological temporal coding features in the form of noise-induced stochastic resonance and dynamical synapses to increase the model’s performance when its parameters are not optimized for a given input. Using the analog XOR task as a simplified convolutional neural network model, this paper demonstrates two key results: (1) SNNs solve the problem that is linearly inseparable in ANN with fewer neurons, and (2) in leaky SNNs, the addition of noise and dynamical synapses compensate for non-optimal parameters, achieving near-optimal results for weaker inputs.

## 1. Introduction

AI training and inference servers’ power consumption is kept at bay with tensor processing units (TPUs), shortening the circuit paths and processing data locally rather than spending energy on transit [1]. However, we are at the limit of how small computer chips can be in order to work reliably, continuing to depend on longer wait times and power draws. Exploring alternative solutions, researchers have shown that spiking neural networks (SNNs) outperform conventional static artificial neural networks (ANNs) [2] in energy efficiency when running models on neuromorphic chips [3], offering a remarkable reduction in energy consumption from 100 times [4] to 280 times [5].

Spiking networks have also shown the ability to self-optimize to changing probability distributions, such as the likelihood of a reward [6] and the adaptability of a dynamic motor control system to a changing environment [7]. This paper shows that noise sensitivity in SNNs with dynamical synapses leads to surprisingly better performance than ANNs when parameters are not fully optimized.

Unlike ANNs, training the synaptic weights of SNNs is challenging due to the instantaneous spiking activity and dynamic synaptic characteristics that hinder gradient computation. Several methods exist to achieve trained SNNs. One method is to obtain synaptic weights by ANN backpropagation and directly apply them to SNN [8]. SNNs can also be trained directly with supervised learning error-based methods, resulting in faster convergence and many possible solutions [9]. Examples include SNN backpropagation [10], hybrid learning of STDP-based unsupervised pre-training and supervised fine-tuning [11], and an approximation of backpropagation with dendritic cortical microcircuits [12]. Other approaches for supervised learning in SNNs are the target propagation methods, which result in a constrained solution [9]. For example, there is a method of target propagation based on the Gauss–Newton optimization [13] and a gradient-free training method for SNN [14].

In order to compare the adaptability of ANN and SNN models, we create two similarly structured networks designed to run the analog XOR function. The goal of the analog XOR task is to check if the inputs are within a certain range of each other; if they are, then the output is low; otherwise, it is high.

The analog XOR task is a non-linear operation and a fundamental building block in neural networks. It is a simple way to analyze the internal processes of networks that can be generalized to aspects of convolutional neural networks, audio processing, and sensor systems [15,16]. Specifically, in both CNN kernels [16] and biological visual processing [17], XOR, in conjunction with logical negation NOT or inhibitory mechanisms, such as lateral inhibition and feedback loops, can approximate functions such as feature extraction and contrast enhancement.

Unlike pioneering ANN-to-SNN network translation research [4,8], our spiking network utilizes temporal coding features: the presence of noise and dynamical synapses.

Noise plays an important role in biological networks, improving the adaptability of a network [18]; it stimulates the effect of stochastic resonance by boosting the near-threshold voltage and generating a spike [19]. Noise also influences the synchronization of inputs and inhibition, which is crucial because the synchronous input is propagated more strongly [19,20]. Noise in biological networks can be attributed to individual neurons and the behavior of a network as a whole. Neuron-specific additive noise is independent of input strength and includes thermal noise and fluctuations in neural membrane conductivity [18,19]. These can be modeled by fluctuating the spike threshold (escape noise), fluctuating any parameter (slow noise), or as an added current (diffusive Gaussian white noise) [19].

The multiplicative noise is proportional to the input and is attributed to the whole network rather than an individual neuron. This noise originates from synaptic transmission failures [19] and is modeled in this work as input-scaled Gaussian white noise. Other architecture-attributed fluctuations originate in fixed random connectivity of neurons [19]. In this work, we compare the effect of additive white noise, additive Ornstein–Uhlenbeck (OU) colored noise [21], and multiplicative white noise on the adaptability of the SNN.

The dynamical synapses regulate the neural connection strength depending on the recent activity: the connection strength increases with recent stimulation and depresses when not activated. In biological networks, dynamical synapses are crucial in implementing plasticity, adaptation, and learning. They enable the detection of temporal patterns, filter high-frequency noise, and drive neural synchronization [19].

The main parameter of the dynamical synapse is the synaptic time constant, $\tau_{syn}$. A higher synaptic time constant means the synapse integrates information over a longer period; a lower $\tau_{syn}$ gives more rapid dynamics. In this work, we look at different values of $\tau_{syn}$. Although parameter tuning (signal gain, $\tau_{syn}$) challenges the convertibility of dynamical synapse network models, we show that the inherent robustness of our spiking network mitigates the need for extensive optimization. The intrinsic adaptability of our spiking network allows it to maintain functionality even with suboptimal parameter configurations.

## 2. Materials and Methods

### 2.1. The Analog XOR Task

We define the analog XOR task for SNNs using the analog input signal $\left(x_{0},x_{1}\right)\in \left[0,1\right]$ and the analog spike count $C^{T}$ within the time frame *T* of the output spiking neuron (N5) as follows:

$C_{N5}^{T}$ will exceed the firing threshold *h* when exactly one of the inputs $\left(x_{0},x_{1}\right)$ is greater than or equal to a boundary value *b* (see the algorithm Table 1).

The final values of the output neuron $C_{N5}^{T}$ are normalized such that *h*∈(0.00, 1.00). The normalization of the final output is used to compare the models, since ANN activations and the SNN spike count are not guaranteed to be proportionally aligned, which depends on other gain and the synaptic time-constant parameters.

The accuracy of the XOR task for an SNN circuit is calculated using the digitized variables $\left(\Theta \left(x_{0}-b\right),\;\Theta \left(x_{1}-b\right)\right)$ and $(\Theta \left(normalized\left(C_{N5}^{T}\right)-h\right)$, where Θ is the Heaviside step function, and Θ(i)=1 and 0 for i>0, and i≤0, respectively.

We find the boundaries (*b*, *h*) and parameters with a grid search. First, we searched for the gain parameters with a fixed boundary on ANN and then tuned the boundary with another grid search of (b, h). One of the best-found boundaries in ANN is (b, h) = (0.46, 0.3) with an 83.74% accuracy. Due to the structure in the output activations of the ANN XOR circuit, many boundaries yield similar accuracy. The same gain parameters did not work for the SNN with $\tau_{syn}$ = 1 ms, and we applied another grid search for parameters given the boundary (b, h) = (0.46, 0.3). The gain parameters that worked best for SNN with $\tau_{syn}$ = 1 are (input gain, weight gain) = (5, 110) or (6, 111). Due to the steps in the grid search and the bias gain parameter set to 1, the returned setting is not guaranteed to be the most optimal.

After finding the optimal boundaries, we observed how the circuit performed with noise added for suboptimal boundaries.

### 2.2. The Circuits’ Structure

**Figure 1:**
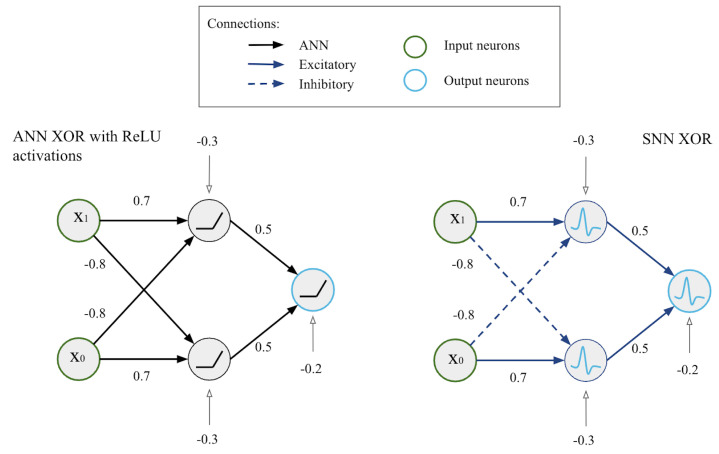
XOR circuit diagrams with initial connection strengths. (**left**): ANN XOR circuit. (**right**): SNN XOR circuit.

The ANN and SNN circuits have two input neurons, two hidden-layer neurons, and one output neuron (Figure 1).

The input and connection weights of the circuits are scaled by the input gain and weight gain; the bias gain is set to 1. The ReLU activation in the ANN circuit is chosen based on previous research of the ANN-to-SNN translation method: ReLU neurons are a good approximation of discrete spikes and are efficient during training [8].

### 2.3. Implementation of Noisy Spiking Neurons

The spiking circuit is simulated with NEST 2.20.1 [22], consisting of leaky integrate-and-fire *iaf_psc_exp* neurons with a dynamical synapse model *tsodyks_synapse* [23] and the parameters listed in Table 2.

We selected the leaky model for this work due to its biological plausibility [19] and higher susceptibility to stochastic resonance compared to a non-leaky neuron. We have tested the firing rate of the non-leaky neuron with static synapse at different noise levels (see Figure S2). Unlike leaky SNN, static non-leaky SNN produced ANN-like output with higher noise intensity. The non-leaky SNN is simulated in C++ with the same parameters.

The leaky neurons’ membrane potential is defined by

(1) $$\tau_{m}\frac{dV_{m}}{dt}=-V_{m}\left(t\right)+I_{bias}+I\left(t\right)+I\left(t\right)\xi^{mul}\left(t\right)+\xi^{add}\left(t\right)+z\left(t\right),$$

where:

- $I_{bias}$ is the base current (18 pA) plus the biases set by the network architecture;
- I(t) is the postsynaptic input current;
- $\xi^{mul}$ and $\xi^{add}$ is Gaussian white noise generated from the probability density function f(x) of normal distribution with 0 mean and standard deviation *D*:
  (2) $$f\left(x\right)=\frac{1}{D\sqrt{2\pi }}e^{-\frac{1}{2}{\left(\frac{x}{D}\right)}^{2}};$$
- z(t) is the colored noise implemented with the Orstein–Uhlenbeck process [21]. z(t) is defined with Gaussian noise ξ(t) and the damping term *a* as
  (3) $$\tau_{z}\frac{dz}{dt}=-az+\xi \left(t\right).$$

The white noise is implemented with the *numpy.random.default_rng().normal* function. It can be applied directly as additive noise ($\xi^{add}$), or it can be scaled by input values to mimic synaptic multiplicative noise ($I\left(t\right)\xi^{mul}$), that is, accumulated voltage that did not generate a spike [19]. Although different types of noise are introduced together in the same Equation (1), in the experiments they were applied individually for comparison.

Unlike unpredictable white noise, the colored noise of the Orstein–Uhlenbeck (OU) process [21] has a non-uniform power spectral density: its values depend on the previous values. The OU process is implemented with the discrete-time approximation of Equation (3) with the damping term (mean-reversion rate) θ=a/τ_z. The mean-reversion rate describes how fast the process converges to the selected mean 0. A lower θ increases the autocorrelation of the process. See Figure S1 for sample noise patterns.

Noise is applied only to the input neurons. This noise can be common (identical) across both inputs or independent (different noise values drawn from the same distribution). A new noise value is sampled every 0.1 ms, which is the temporal resolution of the simulation.

There are fundamental differences in how noise affects the two networks. In the SNN, noise continuously interacts with the network’s dynamics, influencing synchronization and inhibition before the final spike number is counted. However, in the ANN, negative activations are suppressed by the ReLU activation function, and the effect of the noise is mitigated by averaging the output.

## 3. Results

### 3.1. Networks Output Comparison: Non-Linear Separability with Fewer Neurons

**Figure 2:**
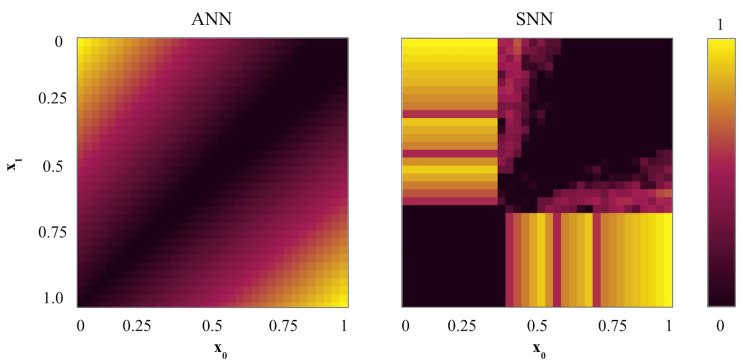
Output example without noise. Parameters: input gain = 6, weight gain = 111. (**left**): Normalized output values in ANN XOR (with ReLU activations). (**right**): Normalized firing frequency in SNN XOR with dynamical synapse $\tau_{syn}$ = 1 ms.

The ANN of the given circuit structure returns the linear separation of the solution field (Figure 2), while the SNN produces a non-linear solution with the same number of neurons.

The SNN XOR can give high-accuracy results at 95% or more with only five neurons. However, this success would only apply to a couple of specific decision boundaries. For example, in Figure 2 (right), we have 95.5% for (b, h) = (0.36, 0.5). The optimal boundary in SNN would greatly depend on the preset parameters: input gain, weight gain, synaptic time constant of a dynamical synapse, and noise level. The linearity of the ANN circuit solution peaks at 83.74% and produces low variability in accuracy for most boundaries.

It is possible to achieve high accuracy (up to 100%) in an ANN circuit for the analog XOR task with a sigmoidal activation function rather than ReLU by applying another layer with a steep sigmoid function to the inputs, which would digitize the analog signal $x_{i}$ to 0 or 1. Insofar as $x_{i}$ is an analog signal between [0, 1], and the ANN structure consists of five neurons as in Figure 1, the ANN XOR recognition accuracy remains low regardless of the activation function. However, the SNN of the similar minimal structure is sufficient for the analog XOR task.

**Figure 3:**
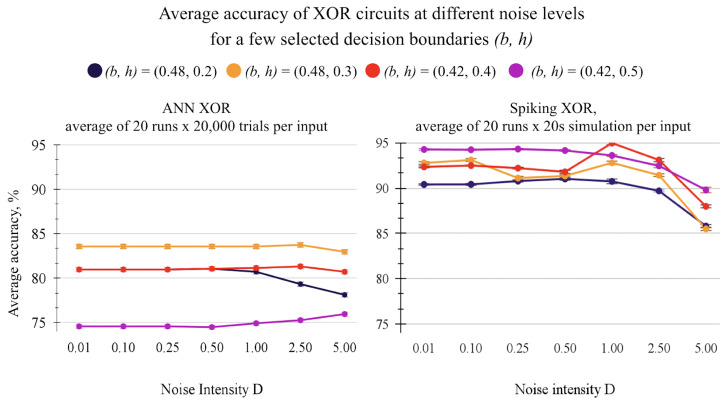
Comparing average accuracy of ANN and SNN circuits for some boundaries across different levels of common multiplicative noise. Tested with input gain = 5, weight gain = 110. (**left**): Non-spiking XOR. (**right**): Spiking XOR with dynamical synapses $\tau_{syn}$ = 1 ms.

Figure 3 shows the accuracy changes with common noise for some selected boundaries. Four boundaries are shown in both graphs: the yellow (b, h) = (0.46, 0.3) is optimal in ANN; the magenta (b, h) = (0.4, 0.5) is the most optimal in SNN; the red (b, h) = (0.42, 0.4) is an example of reaching optimal accuracy with some noise; the dark blue (b, h) = (0.46, 0.2) is a random sample.

Both charts show an average accuracy over 20 runs, where noise values are generated with a new seed every run. In the non-spiking circuit of Figure 3 left, each input is tested with 20,000 different noise components drawn from the same distribution, and then all 20,000 trials are averaged for a single output. The same operation is repeated 20 times for each noise level D to have more samples for the average accuracy result. The spiking circuit (Figure 3 right) is simulated for 20 s per input, with a new noise component added every 0.1 ms (NEST resolution setting), with a total of 200,000 noise components per input. This is repeated 20 times for each noise level D. Since the accuracy is measured on normalized data, the standard deviation of the average accuracy is low.

Figure 3 shows that the current implementation of the circuits gives (1) some improved accuracy for certain boundaries due to the non-linear separation of the output (Figure 2), (2) the noise effect is more dramatic in the SNN than in the ANN, and (3) some boundaries can have the accuracy improved with noise.

### 3.2. Dynamical Synapses in Leaky SNN and Their Effect on Stochastic Resonance

Different from static synapses that have a fixed connection strength, dynamical synapses regulate their conductance based on the previous activity. Increasing the synaptic time constant $\tau_{syn}$ extends the amount of time it takes to decay the synapse strength back to its resting state after a presynaptic spike [23], thus allowing the circuit to integrate input over a longer period.

**Figure 4:**
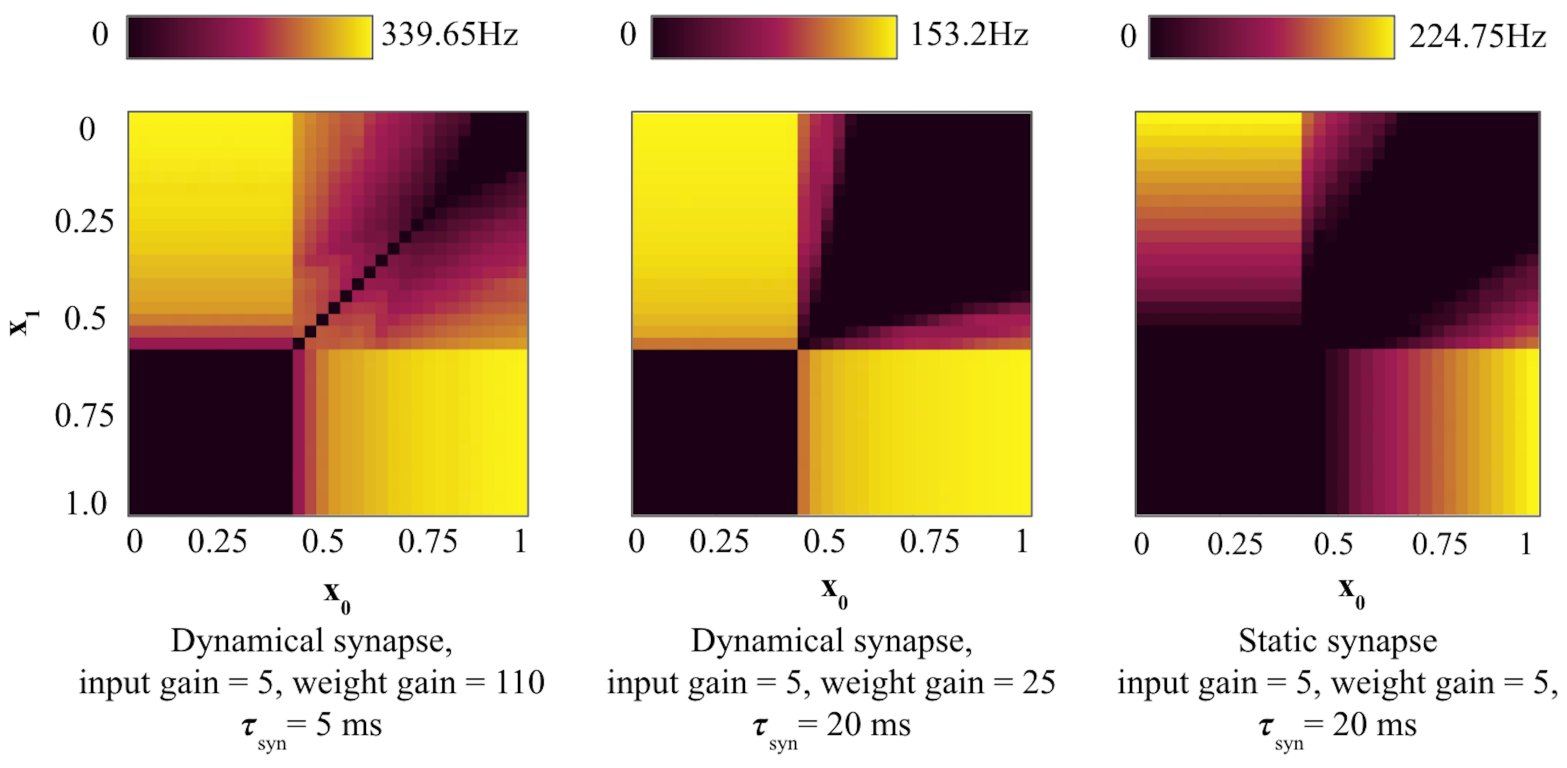
Firing frequency in SNN XOR with different synapse settings. Low intensity D = 0.1 common multiplicative noise is applied. (**left**): Dynamical synapse with $\tau_{syn}$ = 5 ms. (**center**): Dynamical synapse with $\tau_{syn}$ = 20 ms. (**right**): Static synapse.

When $\tau_{syn}$ is high, as in Figure 4 center, the output neuron has a high contrast firing frequency, compared to the lower $\tau_{syn}$ = 1 ms in Figure 2 right and $\tau_{syn}$ = 5 ms in Figure 4 left.

**Figure 5:**
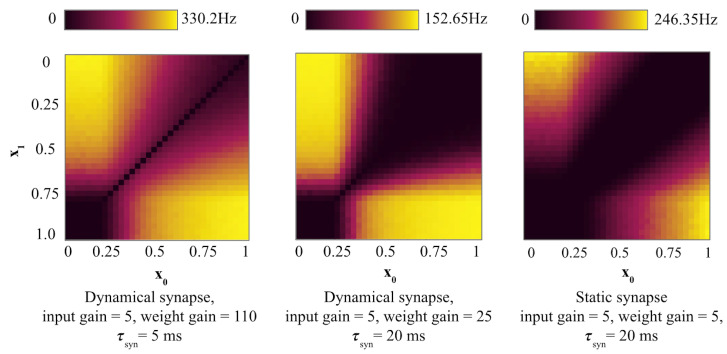
Firing frequency in noisy SNN XOR with different synapse settings. High intensity D = 5 common multiplicative noise is applied. (**left**): Dynamical synapse with $\tau_{syn}$ = 5 ms (**center**): Dynamical synapse with $\tau_{syn}$ = 20 ms. (**right**): Static synapse.

Figure 5 shows the effect of strong common multiplicative noise on the output firing frequency in the SNN with dynamical and static synapses. All synapse configurations show moderate stochastic resonance with a shift in sensitivity to lower boundaries; however, the dynamical synapse with a high synaptic time constant maintains sharper contrast in the firing rates, which would allow it to perform more reliably even at high-intensity noise.

**Figure 6:**
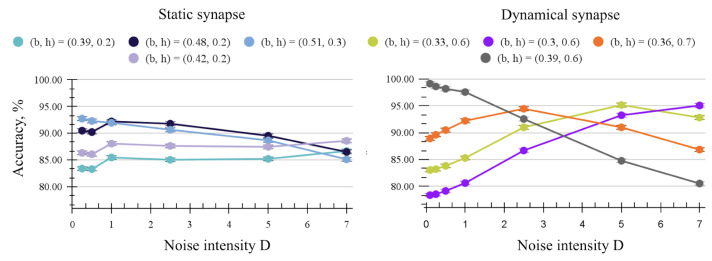
Accuracy of the SNN XOR circuit for best-found boundaries at different common multiplicative noise levels. (**left**): Static synapse, input gain = 5, weight gain = 5. (**right**): Dynamical synapse, input gain = 5, weight gain = 110, $\tau_{syn}$ = 20 ms.

Figure 6 follows the accuracy for some boundaries that are found to be the best at each noise level. Although the static synapse network still exhibits up to 3% improvement attributed to noise for the suboptimal boundaries, the dynamical synapse shows a more measurable and predictable pattern in improving the accuracy for suboptimal boundaries with stronger noise. With dynamical synapses, we have 7% improvement for the more optimal boundary (orange) and 17% improved accuracy for the least optimal boundary (purple).

### 3.3. Improving Accuracy for Sub-Optimal Boundaries in Leaky Dynamic SNN with Noise-Induced Stochastic Resonance

The noise component can be common between both inputs or independent. With common noise, a single value is pulled from the normal distribution and applied to both inputs. Independent noise implies pulling two different noise values from the distribution, one for each input.

The noise component can also be additive or multiplicative. While both additive and multiplicative noise are technically “added” as a fluctuating current, multiplicative noise is scaled by the original inputs and the input gain values; the additive noise is not scaled by gain and is applied uniformly. The multiplicative noise is stronger than the additive noise by a factor of the input gain value in high inputs (which are closer to 1), but the inputs that are closer to 0 generate low noise.

**Figure 7:**
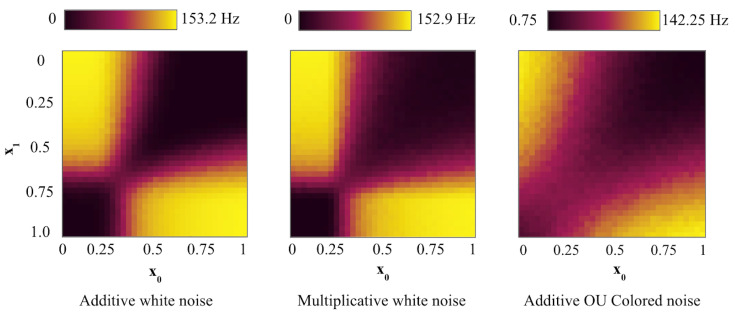
Firing frequency in dynamic SNN XOR with additive and multiplicative noise. Other parameters: input gain = 5, weight gain = 25, $\tau_{syn}$ = 20 ms, noise intensity D = 5. (**left**): Additive independent noise (not scaled by input). (**center**): Multiplicative independent white noise (scaled by input and gain). (**right**): Additive independent OU colored noise, θ=1.

Figure 7 shows the firing rates in networks with different types of strong independent noise. Each noise raises the firing rates in the circuit but at a different rate. Given the same parameters (0 mean, D standard deviation), the OU-colored noise is the most powerful and should be applied at a lower intensity. The multiplicative noise has a stronger shift in sensitivity to the weaker input than the additive noise while maintaining clear non-linear separation.

**Figure 8:**
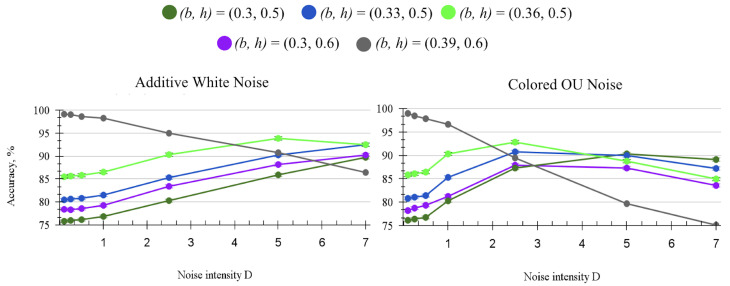
Accuracy of the XOR circuit for some boundaries with different types of noise. Parameters: input gain = 5, weight gain = 25, $\tau_{syn}$ = 20 ms. (**left**): SNN XOR accuracy with independent additive white noise. (**right**): SNN XOR accuracy with additive independent OU colored noise, θ=1.

Figure 8 follows the boundaries that were found to be the most accurate at each intensity of additive (Figure 8 left) and colored noise (Figure 8 right). Both white and colored noise drastically improve the accuracy for the suboptimal boundaries due to stochastic resonance. Although additive noise shows slow improvement with high intensity, colored noise has a much stronger effect with the same standard deviation D, and the accuracy degrades faster after peaking.

**Figure 9:**
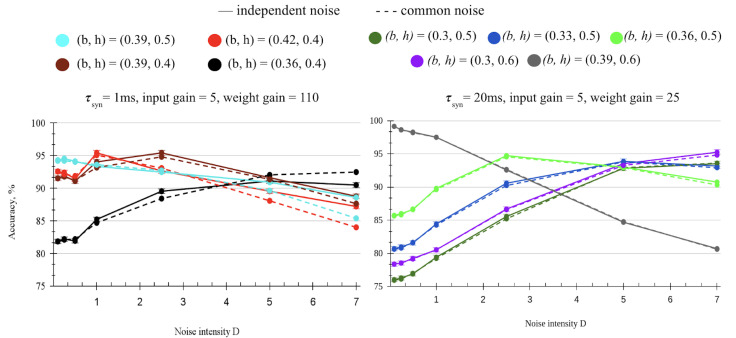
Comparing accuracy of spiking circuit across different levels of common and independent multiplicative noise. The solid line is independent noise, and the dashed line is common noise. (**left**): Parameters: $\tau_{syn}$ = 1 ms, input gain = 5, weight gain = 110. (**right**): Parameters: $\tau_{syn}$ = 20 ms, input gain = 5, weight gain = 25.

Figure 9 right (solid line) follows the same boundaries but with the addition of only multiplicative noise. Both additive and multiplicative noise have a similar effect on the current network. However, compared to additive and colored noise, multiplicative noise reaches near-optimal scores with lower noise and produces higher accuracies per node overall. For example, at D = 5 of multiplicative noise (Figure 9 right), the suboptimal boundaries reach between 92–95% accuracy, the additive noise requires a higher D for similar results, and the colored noise accuracy peaks at 87–93%.

Figure 9 compares the effects of common and independent noise on suboptimal boundaries. The chart shows some boundaries found to be most accurate at each noise level and follows changes in their accuracy. Independent noise gives slightly more improvement than common noise at first, but the trend reverses for less optimal boundaries with high noise intensity (Figure 9 left). Strong common noise is more effective than the independent noise for the boundaries where the model is not optimized, for example, the black boundary (b, h) = (0.36, 0.4).

The accuracy gap between the common and independent noisy networks increases with a higher D as a result of more variation among the individual noise components. This gap is minimal with a high synaptic time constant in Figure 9 right.

Even though the different types of noise have nuances in their effect, we have shown that noise makes the dynamical spiking network more sensitive to weaker input due to stochastic resonance, thus boosting the untuned dynamical spiking network’s performance to near-optimal levels.

## 4. Discussion

In artificial networks, the ability to adapt is another way (besides neuromorphic hardware) to reduce the power consumption during a model’s training and inference. In biological networks, the ability to adapt to changing or missing input is embedded in the brain’s regime of stable propagation of synchronous neural activity among asynchronous global activity [20,24], a combination of rate and temporal coding [25]. The physical properties that contribute to temporal coding are inhibitory connections (feedback) [17], the presence of noise [26], and the short-term plasticity of dynamical synapses [23,27]. These properties are usually omitted from the standard conversion method based on the ANN-to-SNN rate coding [4,8,28]. This work implemented temporal features in the XOR network in the form of multiplicative synaptic noise and dynamical synapses to discuss their effects on adaptation.

The implementation of temporal features in the SNN introduces notable network translation challenges, especially if we seek to apply these results to “convert” existing well-performing ANNs to SNN. The gain parameters for SNN have to be re-selected depending on the synaptic time constant of the dynamical synapse. To make the circuits equivalent and convertible, firing frequency/activations have to be aligned, the ANN activations’ output has to have a similar shape to that of an SNN (non-linear separation), implying an architectural change; a parameter selection rule has to be established, such that the same boundary is returned as the most optimal.

Although ANN models can benefit from noisy training [29], the current set-up showed a minimal noise-induced stochastic resonance effect in ANN. In SNN, noise interacts with neurons’ internal dynamics, and we have more measurable changes with stronger noise when the network is not optimally tuned. We have also shown that the spiking network with multiplicative noise has increased sensitivity to sub-optimal inputs, which can be applied as an adaptation.

The noise types applied in this work show a similar effect, which is likely to be more varied in a larger network. The noisy SNN stochastic resonance presented in this work implies that only the suboptimal boundaries that can be helped by amplifying the firing frequency will be improved. This poses a question for implementing noise with inverse effects that would weaken the firing frequency in stronger inputs and adjust the circuit for higher boundaries instead.

The functionality of a noisy SNN XOR could also be expanded to other functions by implementing other temporal characteristics in the form of inhibitory neuron pools [30], feedback loops, or taking into account the synchronization index [25].

## Figures and Tables

**Table 1:** Algorithm table.

$x_{0}$	$x_{1}$	Normalized $C_{N5}^{T}$
<*b*	<*b*	<*h* (LOW)
<*b*	≥*b*	≥*h* (HIGH)
≥*b*	<*b*	≥*h* (HIGH)
≥*b*	≥*b*	<*h* (LOW)

**Table 2:** *iaf_psc_exp* neuron, *tsodyks_synapse* dynamical synapse, and simulation parameters.

Code Variable	Description, Units	Value
C_m	Membrane capacitance, pF	10.0
tau_m	Membrane time constant, ms	10.0
E_L	Resting membrane potential, mV	0.0
V_m	Initial membrane potential, mV	0.0
V_reset	Reset potential of the membrane, mV	16.0
V_min	Lowest value for the membrane potential	−1.798 $\times \;10^{308}$
refractory_input	Discard input during refractory period	False
I_e	Constant input current, pA	18.0 + bias
V_th	Spike threshold, mV	20.0
tau_fac	Synapse facilitation time constant, ms	1
tau_rec	Synapse recovery time constant, ms	10
tau_syn	Synaptic time constant ($\tau_{syn}$), ms	1
t_sim	Simulation resolution, ms	0.1
T	Simulation time, s	20.0

## Data Availability

The raw data supporting the conclusions of this article will be made available by the authors on request.

## Supplementary Materials

The following supporting information can be downloaded at https://www.mdpi.com/article/10.3390/e27030219/s1, Figure S1: Sample noise values for one hundred time intervals; Figure S2: Relative firing rate in non-leaky SNN XOR at different intensity levels of common multiplicative white noise.
