# Supplementary material for: Noise and Dynamical Synapses as Optimization Tools for Spiking Neural Networks
Source: Entropy (Basel). 2025 Feb 21;27(3):219. doi: 10.3390/e27030219 (PMC11941097; doi:10.3390/e27030219)
Supplement: Supplementary file 1 [file entropy-27-00219-s001.zip › entropy-3465088-supplementary.pdf]

Supplementary Materials:

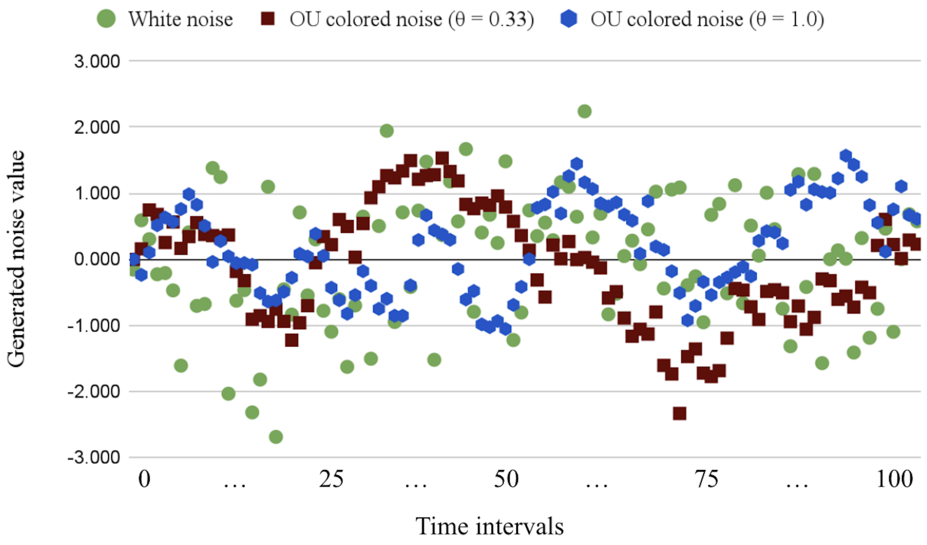

Figure S1. Sample noise values for one hundred time intervals. Green dots are white noise data points distributed according to random normal distribution with 0 mean and standard deviation D = 1. Brown dots are the Orstein–Uhlenbeck (OU) process data points with a low mean-reversion rate θ, 0 mean and standard deviation D = 1. Blue dots are the Orstein–Uhlenbeck (OU) process data points with a high mean-reversion rate θ, 0 mean and standard deviation D = 1.

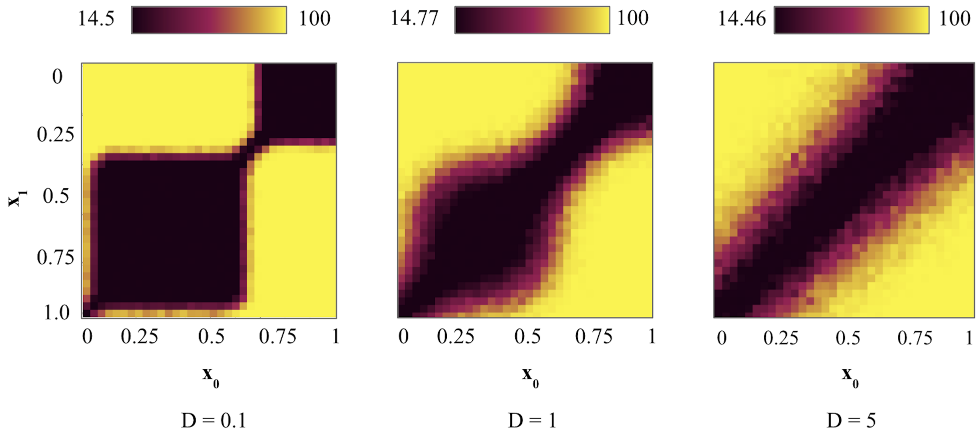

Figure S2. Relative firing rate in non-leaky SNN XOR at different intensity levels of common multiplicative white noise. This non-leaky SNN XOR is built with static synapses, input gain = 5, weight gain = 25. **(left):** Low noise, D = 0.1 **(center):** Middle-strength noise, D = 1. **(right):** High noise, D = 5.0
